# Telmisartan anti‐cancer activities mechanism through targeting N‐cadherin by mimicking ADH‐1 function

**DOI:** 10.1111/jcmm.17259

**Published:** 2022-02-27

**Authors:** Marjan Khorsand, Sahar Khajeh, Mahboobeh Eslami, Navid Nezafat, Younes Ghasemi, Vahid Razban, Zohreh Mostafavi‐Pour

**Affiliations:** ^1^ Department of Biochemistry School of Medicine Shiraz University of Medical Sciences Shiraz Iran; ^2^ Bone and Joint Diseases Research Center Shiraz University of Medical Sciences Shiraz Iran; ^3^ Pharmaceutical Sciences Research Center Shiraz University of Medical Sciences Shiraz Iran; ^4^ Department of Pharmaceutical Biotechnology School of Pharmacy Shiraz University of Medical Sciences Shiraz Iran; ^5^ Molecular Medicine Department School of Advanced Medical Sciences and Technology Shiraz University of Medical Sciences Shiraz Iran; ^6^ Stem Cell Technology Research Center Shiraz University of Medical Sciences Shiraz Iran; ^7^ Autophagy Research Center Shiraz University of Medical Sciences Shiraz Iran

**Keywords:** ADH‐1, cancer, cell attachment, docetaxel, N‐cadherin, telmisartan

## Abstract

This study aimed to investigate if Telmisartan as a novel N‐cadherin antagonist, can overcome cell migration of cancer cells. We investigated the mechanism and influence of Docetaxel and Telmisartan (as an analogous to ADH‐1, which is a well‐known N‐cadherin antagonist) on cancer cells. The effect of ADH‐1 and Telmisartan on cell attachment in PC3, DU145, MDA‐MB‐468 cell lines using recombinant human N‐cadherin was studied. Cell viability assay was performed to examine the anti‐proliferative effects of Telmisartan, ADH‐1 and Docetaxel. Migration was examined via wound healing assay, and apoptosis was determined by flow cytometry. The expression of AKT‐1 as a downstream gene of N‐cadherin signalling pathway was assayed by real‐time PCR. Treatment of PC3, MDA‐MB‐468 and DU145 cells with Telmisartan (0.1 µM) and ADH‐1 (40 µM) resulted in 50%, 58% and approximately 20% reduction in cell attachment to N‐cadherin coated plate respectively. It shows reduction of cell attachment in PC3 and MDA‐MB‐468 cell lines appeared to be more sensitive than that of DU145 cells to the Telmisartan and ADH‐1 treatments. Telmisartan (0.1 µM) and Docetaxel (0.01 nM) significantly reduced cell migration in PC3 and MDA‐MB‐468 cell lines compared with the control group. Using Real‐time PCR, we found that Telmisartan, Docetaxel and ADH‐1 had significant influence on the AKT‐1 mRNA level. The results of the current study for the first time suggest that, Telmisartan, exerts anti‐proliferation and anti‐migration effects by targeting antagonistically N‐cadherin. Also, these data suggest that Telmisartan as a less expensive alternative to ADH‐1 could potentiate Docetaxel anticancer effects.

## 1 INTRODUCTION

Prostate cancer (PCa) is one of the most common solid tumour and globally the frequent cause of cancer‐related death in men, after lung cancer.^1^, ^2^ Chemotherapy by docetaxel (DTX) is commonly used to treat a broad range of human malignancies, including lung cancer, breast, head and neck, stomach and PCa.^3^ DTX belongs to the taxanes class and is the standard first‐line treatment against metastatic PCa; however, its use for long term, results in many side effects and drug‐resistance. Therefore, exploring new drugs with less side effects and potential to inhibit migration and metastasis of malignant cells is valuable.^4^

PCa commonly metastasizes to organs such as liver, brain, lymph nodes and bone, which is one of the major causes of deaths related to this malignancy.^5^ This process involves several steps, including cell‐extracellular matrix and cell‐cell attachments and detachments that allows the invasive cells disseminate from the primary tumour and migrate to the other organs.^6^ In normal epithelium, cell‐cell adhesion is maintained by many distinct junctions, such as tight junctions, which are composed of the complexes of cell adhesion molecules (CAMs), including selectin and cadherin proteins.^7^ Epithelial to mesenchymal transition (EMT) is often associated with loss of E‐cadherin expression level and increased the level of N‐cadherin expression, leading to enhanced cell migration and invasiveness in various types of cancers.^7^ Many studies have indicated that EMT plays a fundamental role in tumours development and progression, suggesting that new drugs targeting EMT and its mediators might be effective against different cancers. Many small molecules have been established for inhibiting EMT by targeting the mediators, such as the commercial drugs and nature‐based compounds. Small molecules, which is composed about 75% of the anticancer FDA‐approved drugs, have an extended history of using as drugs for the treatment of many diseases including diabetes, hypertension, infections, heart failure, rhinitis and cancers.^8^, ^9^

Previous study reported a novel peptide, ADH‐1, that inhibit N‐cadherin (a marker of EMT) function.^10^ Yarom et al.^11^ showed that ADH‐1 was commonly well tolerated up to a dose of 1000 mg/m^2^ when administered every three weeks in patients with incurable solid tumours. Other researchers demonstrated that significant reduction in tumour growth and lung metastasis by ADH‐1 in a mouse model of pancreatic cancer.^12^ Although ADH‐1 has high specificity and tolerance, the oral administration of it, as the most convenient and comfortable way, is a difficult challenge for this drug. Based on the Lipinski's Rules, peptide and protein drugs are difficult to passing through the gastrointestinal mucosa and easily degraded by gastric acid, and resulting in poor bioavailability of oral delivery (less than 1%). Currently, most peptide and protein drugs are administered by invasive techniques such as subcutaneous or intravenous (IV) injection that require professional medical personnel.^13^ Compared with peptides, the small molecules dominate the global drug market due to their advantages such as small size, oral availability, low cost, membrane‐penetrating ability, ready synthesis and stability.^14^ Therefore, the use of new drugs with less invasiveness administration route, controllability and high bioavailability is very efficient.^13^ In previous *in silico* studies, after deep analysis of N‐cadherin/ADH‐1 interaction, we screened for FDA‐approved small molecules which can act as potential inhibitors for N‐cadherin protein.^15^, ^16^ Among seven screened candidate drugs, we decided to use one of these inhibitors, Telmisartan (Tel) in the current study. Our hypothesis was that Tel prevents cell migration and inhibits attachment of PCa and breast cancer cells to recombinant N‐cadherin coated wells by suppressing N‐cadherin. Tel is a specific and selective blocker for angiotensin II receptor, which is widely utilized as an antihypertensive drug and recently have been used for their anticancer effects.^17^ Moreover, studies have demonstrated that Tel can inhibit the proliferation and growth of tumour cells. Moreover, it possess cytotoxic effects on different cancer cells when prescribed alone or in combination with other antitumor drugs.^18^ To the best of our knowledge, no study has reported the effects of Tel on N‐cadherin‐mediated cell migration yet. Therefore, the aim of the present study was to investigate the potential anticancer effects of ADH‐1, as a known N‐cadherin antagonist, DTX, as the first line chemotherapy agent in PCa treatment, and Tel, as a novel small molecule, on cell viability, apoptosis, migration and cell attachment. In previous studies, the anticancer effects of Tel have been reported^18^, ^19^, ^20^, ^21^; so, in the current study, in addition to the N‐cadherin antagonist function, we decided to investigate the anticancer effects of Tel in comparison to the first line chemotherapy drug for treatment of prostate cancer, DTX.

N‐cadherin expression and homophilic ligation of N‐cadherins between adjacent cells by activation of PI3K/AKT signalling pathway promotes growth regulatory events which was associated with angiogenesis, metastasis, programmed cell death and EMT in various cancers.^22^, ^23^ Therefore, the effect of aforementioned drugs on AKT‐1 gene expression as a downstream gene of N‐cadherin signalling was also investigated.

The two main types of prostate cancer cell lines (PC3 and DU145) and a breast cancer cell line (MDA‐MB‐468) were used for the present study. DU145 cell line lacks the N‐cadherin expression, while PC3 and MDA‐MB‐468 cell lines highly express N‐cadherin.^24^ We evaluated the response of each cell lines to the aforementioned drugs.

## 2 MATERIALS AND METHODS

### 2.1 Materials

Recombinant human N‐cadherin protein was obtained from R&D Systems. DTX, ADH‐1 and Tel were obtained from MedChem Express. All drugs were prepared in dimethyl sulfoxide (DMSO; Sigma‐Aldrich) and diluted with media before each experiment. RPMI‐1640 medium, fetal bovine serum (FBS) and penicillin–streptomycin were purchased from Invitrogen Life Technologies (Gibco, Life Technology).

### 2.2 Cell culture

The PC3, DU145 (Prostate cancer) and MDA‐MB‐468 (Breast cancer) cell lines were obtained from the National Cell Bank of Iran (Pasteur Institute, Iran). The cells were cultured with RPMI‐1640 medium supplemented with 10% FBS, 1% penicillin–streptomycin antibiotic solution and maintained in humidified incubator with 5% CO_2_ at 37°C. All experiments were carried out at passage number 2–4 after reaching 70% confluency.

### 2.3 Cell viability assay

The viability of PC3, DU145 and MDA‐MB‐468 cells was determined using 3‐(4,5‐dimethylthiazol‐2‐yl)‐2,5‐diphenyltetrazolium bromide (MTT) assay.^25^ Briefly, 2 × 10^4^ of PC3, DU145 and MDA‐MB‐468 cells were seeded into 96‐well plate and cultured for 24 hours. Then, the cells were treated with various concentration of DTX (0.001–100 nM), ADH‐1 (0.001–500 µM), Tel (0.001–500 µM) for 48 hours. Thereafter, MTT solution (contains 0.5 mg/mL MTT) was added to each well. After incubation at 37°C and dissolution the formazan crystals in DMSO, absorbance of each well was measured at 570 nm using a microplate reader (Mikura Ltd.). Percent cell viability was determined with respect to the control. All concentrations were tested in quadruplicate wells and the experiment was repeated three times.

### 2.4 Cell migration assay

Monolayer wound healing assay was used to determine cell migration according to described protocols.^26^ PC3, DU145 and MDA‐MB‐468 cells were seeded on 24‐well plate and incubated at 37°C to grow about 70%–80% confluency. A vertical scratch was created by a plastic sterile 10 μL pipette tip followed by washing the cells with 1X PBS (phosphate buffer saline) to remove the loose cells and debris. Then, cell culture media (for control cells) or media contain the drugs (for treated cells) were added, and the plates were incubated in a CO_2_ incubator at 37°C. All wounds were photographed in three different locations with 40X magnification using a MicroOptix microscope at zero‐time point and by interval of 6 hours over a span of 24 hours after treatment. All images were taken with a camera (Microscopy vision, CAMV1200SC, Canada) equipped with vision capture software. The experiments were repeated three times, and each experiment was performed in triplicate wells. Because the created wound in the control group of PC3 cells completely closed after 24 hours, the DU145 and MDA‐MB‐468 cells were also examined at the end of 24 hours. The area of each wound was measured at each time point using Image J software (NIH**),** and the percentage of wound closure was calculated compared with the control cells.

### 2.5 Cell attachment assay

Cell attachment assay was carried out in the adhesive 96‐well plates as described previously.^27^ Briefly, the wells were coated with 5 µg/mL of recombinant human N‐cadherin, and then, 50 μL of cell suspension (4 × 10^5^/mL) were loaded to each coated well together with 50 μL of drugs diluted with cell culture medium and incubated in 37°C. In the next step, unbound cells were removed, and the adherent cells were fixed by glutaraldehyde. The fixed cells were stained with crystal violet (0.1% (w/v) in methylethanesulphonic acid (MES)), and finally, the optical density of each well was measured with a microplate reader at 570 nm.

### 2.6 Reverse transcription‐quantitative polymerase chain reaction (RT‐qPCR)

Total RNA was extracted from cells by the Tripure reagent, and concentration of RNA was determined by a Nanodrop 1000 spectrophotometer. RNA was converted to cDNA using the cDNA synthesis kit (Fermentas; Thermo Fisher Scientific, Inc.), based on the manufacturer's protocols. Quantitative real‐time PCR (qPCR) was performed by the SYBR Green PCR Master Mix with the following set of specific primers: GUSB‐β‐Glucuronidase – (as an internal control); forward: 5′‐TCGCTCACACCAAATCCTT‐3′, reverse: 5′‐GGCTTCTGATACTTCTTATACCA‐3′ and AKT‐1; forward: TTGTTATTGTGTATTATGTTGTTCA, reverse: AAGTGCTACCGTGGAGAG. qPCR amplification was performed for 95°C for 10 minutes, then 40 cycles using the following program: 95°C for 15 seconds, 60°C (AKT‐1) or 58°C (GUSB) for 30 seconds, 72°C for 30 seconds. Data analysis was carried out using Rotor‐Gene Q Series Software. The expression of AKT‐1 gene was normalized to the level of GUSB expression within each sample, and the relative expression of AKT‐1 was calculated using Pfaffl method as described previously.^28^

### 2.7 Apoptosis assay by Flow Cytometry

The status of cell death was measured by flow cytometry method. Briefly, 4 × 10^5^ cells were seeded in 6 well plates and incubated with drugs (treated cells) or cell culture medium (control cells) for 48 hours. In the next step, the cells were detached using cell dissociation buffer (non‐enzymatic) and were washed twice with PBS. Then, the cells were stained with Annexin‐V conjugate (FITC) and propidium iodide (PI) (MabTag). After incubation for 20 minutes in the dark, the population of apoptotic cells was analysed by flow cytometry (BD FACS Calibrator, Biosiences Company). Each experiment was repeated by three independent experiments and the results analysed by FlowJo 7.6 software.

### 2.8 Statistical analysis

Data in our study were collected from at least three independent experiments. Statistical analysis was determined using One‐way analysis of variance (ANOVA) with Tukey post hoc test for multiple comparisons using SPSS software (version 22.0). All graphs and calculation of IC_50_ values were performed using GraphPad Prism 6.0 software (GraphPad Software). Data were expressed as mean ± SD, and values with *p* < 0.05 were considered statistically significant.

## 3 RESULTS

### 3.1 The cytotoxic effects of DTX, Tel and ADH‐1 against prostate and breast cancer cell lines

**FIGURE 1:**
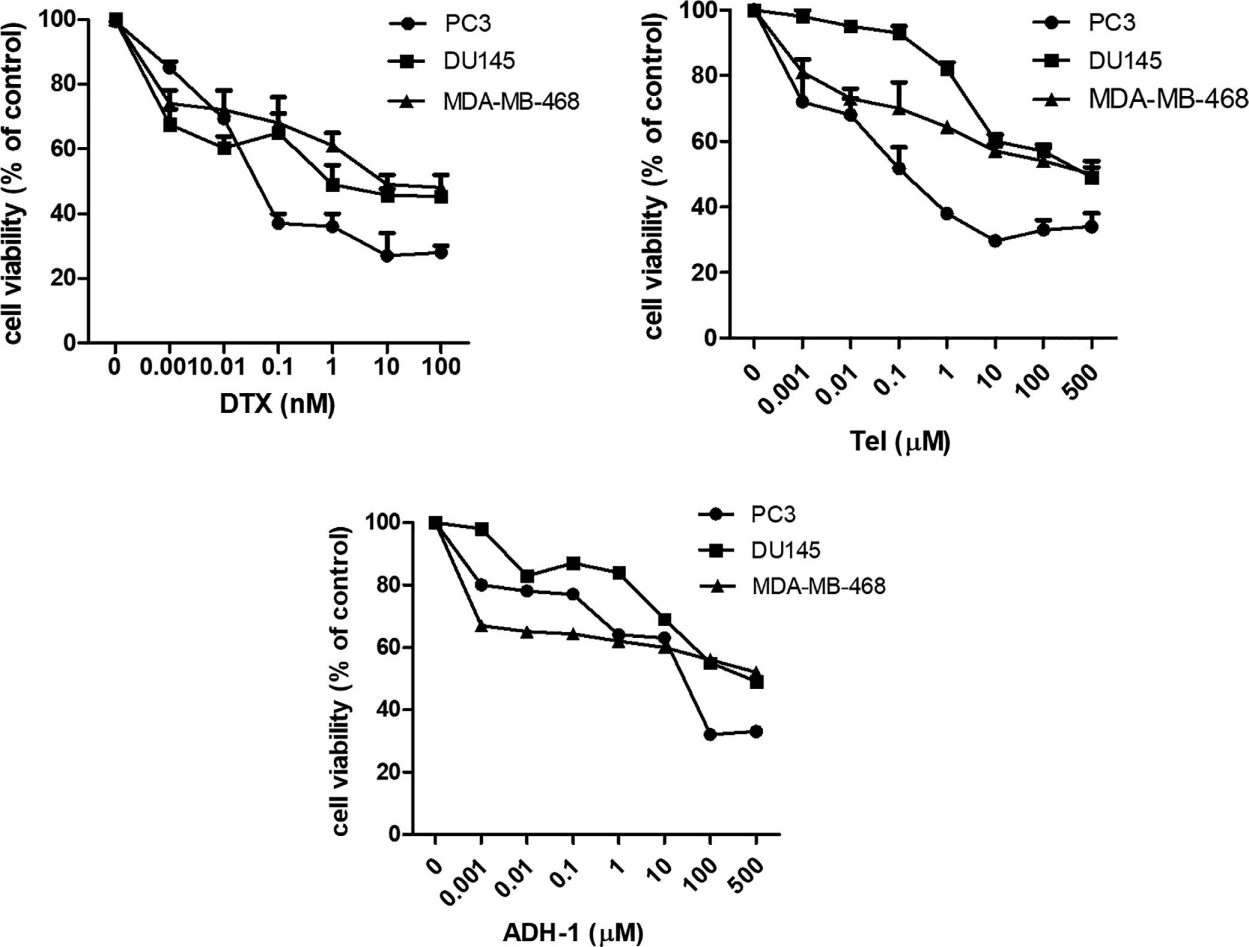
Effect of DTX, Tel and ADH‐1 on viability of prostate (PC3, DU145) and breast cancer (MDA‐MB‐468) cell lines. The cells were treated with different doses of DTX (0.001–100 nM), Tel and ADH‐1 (0.001–500 µM) for 48 hours. The control cells were treated only with the cell culture medium. Data are expressed as the mean ±SD of three independent experiments (n = 12). DTX; Docetaxel, Tel; Telmisartan

The MTT assay was carried out for evaluation of cell proliferation at 48 hours time point, and with different concentrations of DTX (0.001–100 nM), Tel and ADH‐1 (0.001–500 μM). The results indicated that all drugs could inhibit PCa and breast cancer cell proliferation in a dose‐dependent manner (Figure 1, *p* < 0.001). DTX, Tel and ADH‐1 decreased the proliferation of PC3 cells with IC_50_ of 0.02 nM, 0.10 µM and 30.70 µM respectively. In addition, DTX, Tel and ADH‐1 decreased the viability of DU145 cells with IC_50_ of 0.66 nM, 1.68 µM and 7.4 µM and decreased the viability of MDA‐MB‐468 cells with IC_50_ of 1.03 nM, 0.45 µM and 1.85 µM respectively, as shown in Table 1.

**TABLE 1:** IC_50_ values of DTX, Tel and ADH‐1 in different cancer cell lines

PC3	DU145	MDA‐MB468	IC_50_
0.022 (95% CI: 0.008–0.062)	0.665 (95% CI: 0.054–8.16)	1.038 (95% CI: 0.361–2.981)	DTX (nM)
0.101 (95% CI: 0.048–0.228)	1.684 (95% CI: 0.870–3.25)	0.454 (95% CI: 0.024–8.48)	Tel (µM)
30.700 (95% CI: 0.136–69.16)	7.400 (95% CI: 0.037–14.45)	1.853 (95% CI: 0.073–46.6)	ADH‐1 (µM)

Note

Data are expressed as the mean ± standard deviation of three independent experiments (n = 12).

Abbreviations: CI, confidence interval; IC_50_, 50% inhibitory concentration.

### 3.2 Effects of DTX and Tel on cell migration in prostate and breast cancer cell lines

**FIGURE 2:**
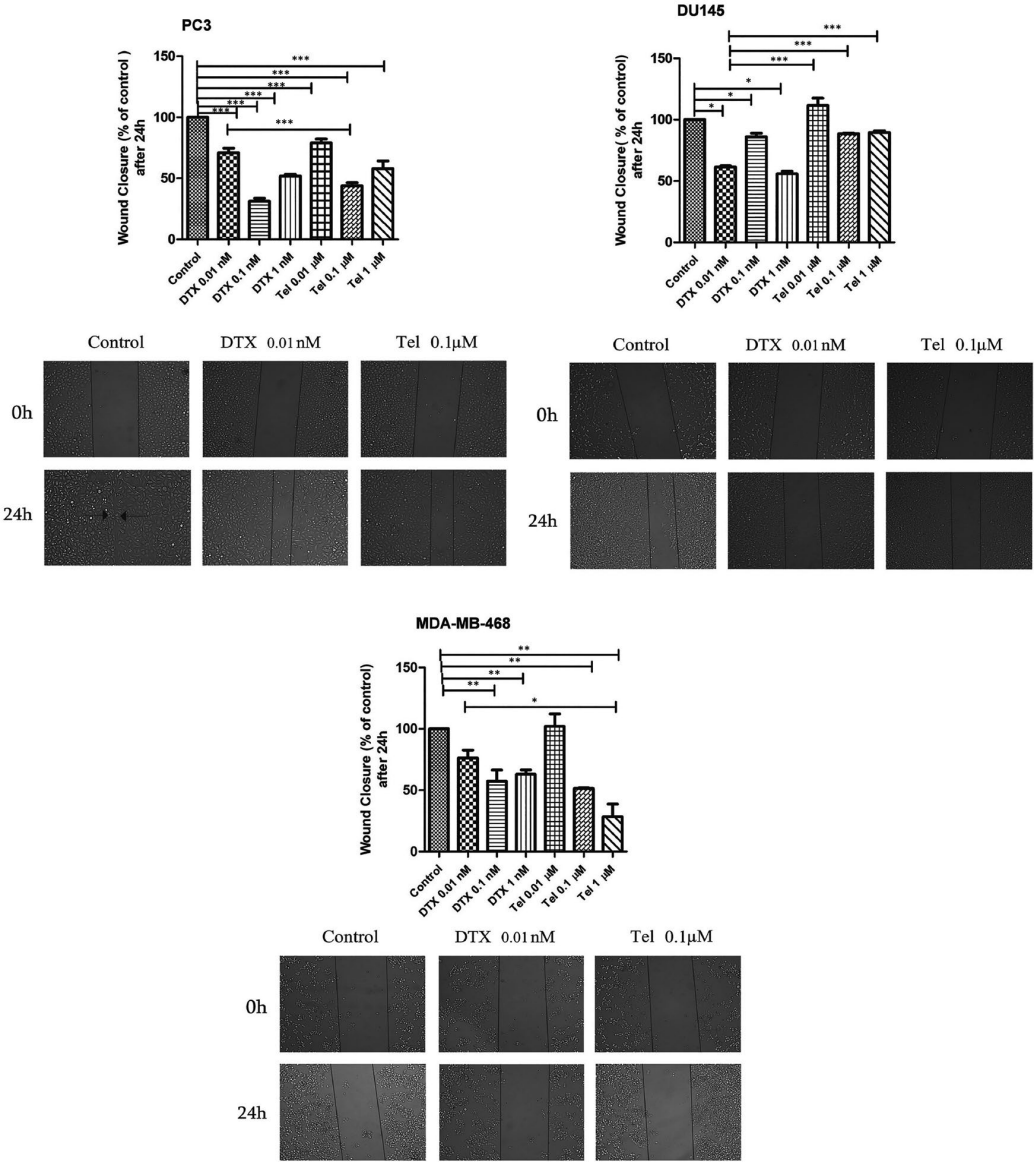
Effect of DTX and Tel on migration of prostate and breast cancer cell lines. The scratch wound was created using 10 µl sterile pipette in 70% confluency of PC3, DU‐145 and MDA‐MB‐468 cells. Media containing certain concentration of each drug was added to defined wells, and the images were taken at 0 and 24 hours by an inverted microscope (×40 magnification). The lines show the area where the scratch wound was created. The scratch wound assay was performed in triplicate wells for each concentration. Data are expressed as the mean ±SD of three independent experiments (n = 9). DTX; Docetaxel, Tel; Telmisartan. *p < 0.05, ** *p* < 0.01, ***p < 0.001

To evaluate the effects of DTX and Tel on metastatic potential of PC3, DU145 and MDA‐MB‐468 cell lines, *in vitro* wound healing assay was performed. We found that the PC3 cells which treated with different concentrations of DTX and Tel after 24 hours obtained slower closure of the wound significantly to compare with untreated cells (*p* < 0.001). As indicated in Figure 2, the inhibitory effect of Tel (0.1 µM) on PC3 cell migration was more than DTX (0.01 nM, *p* < 0.001). Moreover, Tel (0.01 µM) and DTX (0.01 nM) have the same inhibitory effects on cell migration. Treatment of DU145 cells with DTX (0.01–0.1–1 nM) could decrease cell migration after 24 hours significantly compared with untreated cells (*p* < 0.001), whereas Tel (0.01–0.1–1 µM) has no significant inhibitory effect on DU145 cell migration (Figure 2). Besides, DTX (0.01 nM) significantly decreased cell migration in DU145 cells compared with different doses of Tel (*p* < 0.001). Moreover, DTX (0.1, 1 nM) and Tel (0.1, 1 µM) could decrease the migration of MDA‐MB‐468 cells compared to untreated cells after 24 hours (*p* < 0.001). In addition, Tel 1 µM was more effective on reduction of wound closure rate compared to DTX 0.01 nM in MDA‐MB‐468 cells. As seen in Figure 2, the effects of various concentrations of DTX and Tel on cell migration were examined in all three cell lines. Based on these results and IC_50_ values of both drugs, subtoxic concentrations of DTX (0.01 nM) and Tel (0.1 μM) were selected for subsequent experiments.

### 3.3 Effects of Tel and ADH‐1 on cell attachment in prostate and breast cancer cell lines

**FIGURE 3:**
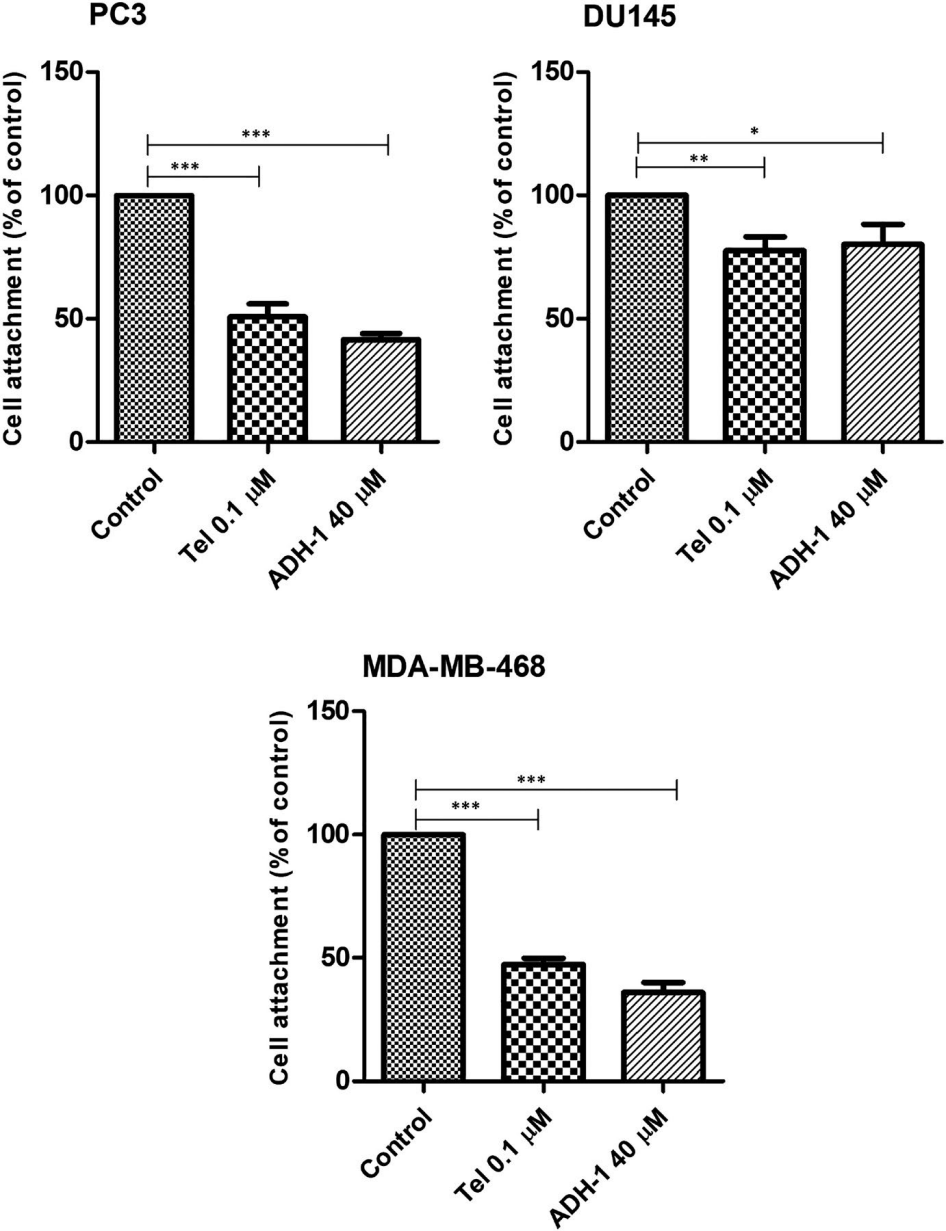
Effect of Tel 0.1 μM and ADH‐1 40 μM on PC3, DU145 and MDA‐MB‐468 cell attachment to recombinant human N‐cadherin (5 μg/mL). The level of nonspecific binding, determined from these cells attachment to wells coated with BSA alone, was subtracted. Values are mean ±SD of three independent experiments. Results show representative of repeated experiments and normalized to 100% binding for untreated cells as control. Tel, Telmisartan. *p < 0.05, ** *p* < 0.01, ***p < 0.001

To clarify the effect of Tel and ADH‐1 on cell attachment in PCa and breast cancer cell lines, we examined the adherence of cells treated with the various concentration of drugs (Tel, ADH‐1) to N‐cadherin‐coated plate (data not shown), and then, the effective doses were selected. According to many evidences, the function of DTX is through inhibition of microtubule depolymerisation and has no specific effect on N‐cadherin function and cell attachment; therefore, in this experiment only the effects of Tel and ADH‐1 were investigated. Treatment of PC3 and MDA‐MB‐468 cells with Tel, 0.1 µM, and ADH‐1, 40 µM, resulted in a significant decrease in cell attachment compared with the controls (*p* < 0.001). PC3 cells treated with 0.1 µM of Tel resulted in 50% reduction in cell attachment (Figure 3, *p* < 0.001) while, 40 µM of ADH‐1 resulted in 58% reduction in cell attachment. In addition, MDA‐MB‐468 cells treated with the same concentrations of Tel and ADH‐1 as abovementioned, resulted in 53% and 64% reduction in cell attachment respectively (Figure 3, *p* < 0.001), whereas, the incubation of DU145 cells with 0.1 μM of Tel and 40 μM of ADH‐1 resulted in 23% and 20% reduction in cell attachment (*p* < 0.05). The attachment of PC3 and MDA‐MB‐468 cells to N‐cadherin appeared to be more sensitive than that of DU145 cells in the presence of Tel and ADH‐1.

### 3.4 Effects of DTX, Tel and ADH‐1 on AKT‐1 expression level in prostate and breast cancer cell lines

**FIGURE 4:**
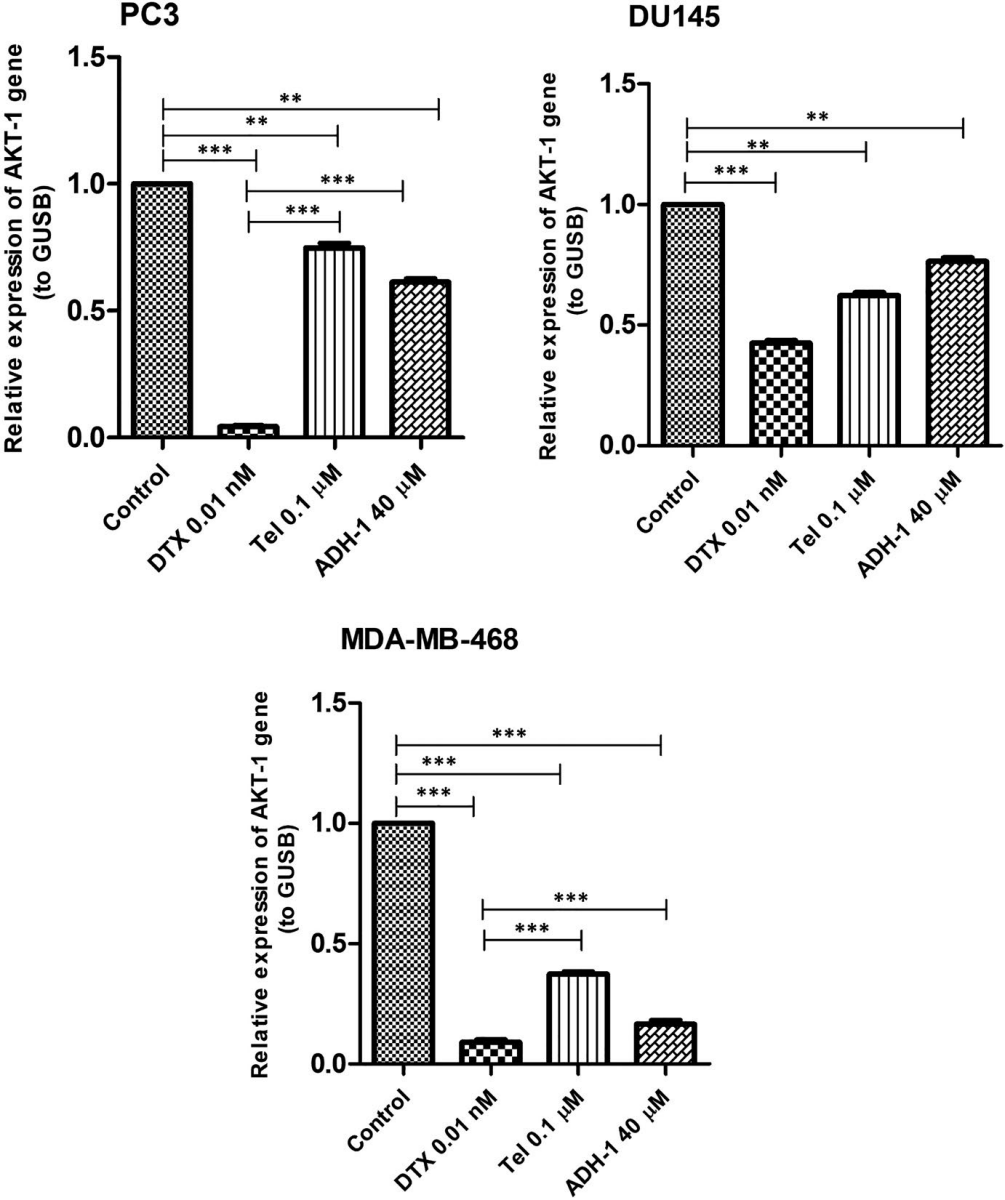
Effect of DTX, Tel and ADH‐1 on AKT‐1 mRNA expression following 48 hours of treatment of PC3, DU145 and MDA‐MB‐468 cell lines that performed by RT‐qPCR. Level of AKT‐1 mRNA expression in various cell lines following treatment with DTX (0.01 nM), Tel (0.1 µM) and ADH‐1 (40 µM) relative to the controls. Each experiment was performed in three independent times. GUSB (β‐Glucuronidase) was used as the loading control. RT‐qPCR, reverse transcription‐quantitative polymerase chain reaction; mRNA, messenger RNA; DTX, Docetaxel; Tel, Telmisartan. *p < 0.05, ** *p* < 0.01, ***p < 0.001

As shown in Figure 4, treatment of PC3, DU145 and MDA‐MB‐468 cells with different concentrations of DTX, Tel and ADH‐1 had a significant decrease in the expression level of AKT‐1 gene compared with control cells (*p* < 0.001). In PC3 cells, DTX, Tel and ADH‐1 could decrease the expression level of AKT‐1 by 4%, 70% and 61% respectively (Figure 4, *p* < 0.001). Treatment of DU145 cells with DTX, Tel and ADH‐1 could decrease the expression level of AKT‐1 by 42%, 62% and 76%, respectively (Figure 4, *p* < 0.01), whereas, DTX, Tel and ADH‐1 decreased the expression level of AKT‐1 by 9%, 37% and 16% compared to control cells, respectively, in MDA‐MB‐468 cells (*p* < 0.001). As seen in Figure 4, treatment of PC3 and MDA‐MB‐468 cells with 0.01 nM of DTX had more effect on the expression level of AKT‐1 compared with 0.1 µM of Tel and 40 µM of ADH‐1 (*p* < 0.001). In addition, the level of AKT‐1 expression in MDA‐MB‐468 cells appeared to be more sensitive to Tel and ADH‐1 than other cell lines.

### 3.5 Effects of DTX, Tel and ADH‐1 on induction of apoptosis in prostate and breast cancer cell lines

**FIGURE 5:**
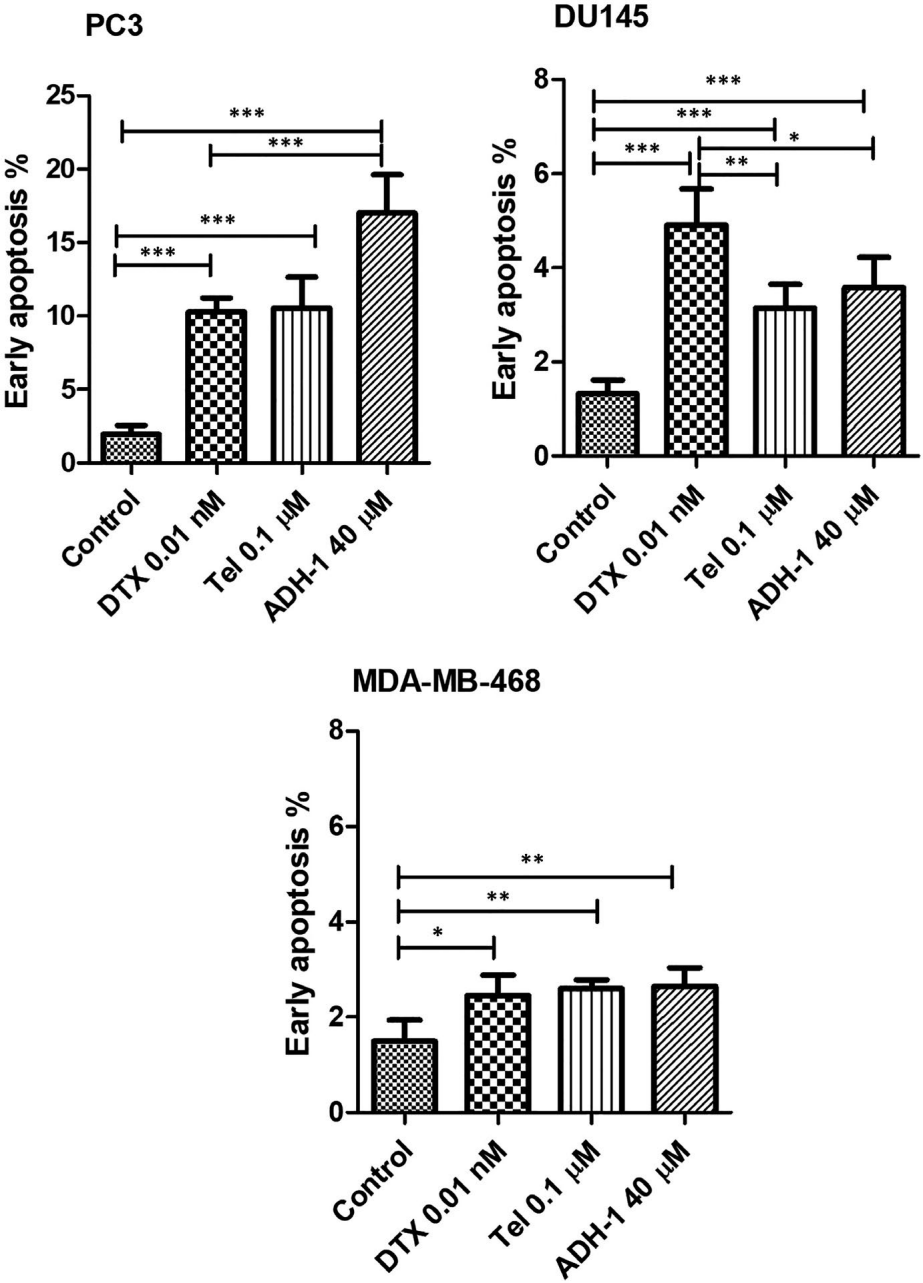
Effect of DTX, Tel and ADH‐1 on cell apoptosis following 48 hours treatment of PC3, DU145 and MDA‐MB‐468cell lines that performed by flow cytometry. Percentage of early apoptosis in various cell lines following treatment with DTX (0.01 nM), Tel (0.1 µM) and ADH‐1 (40 µM). Each experiment was performed in three independent times. DTX, Docetaxel; Tel, Telmisartan. *p < 0.05, **p < 0.01, ***p < 0.001

In order to find out if the Tel and ADH‐1 treatment also induces cellular apoptosis compared with the DTX one, flow cytometry analysis was performed. The exposition of PC3 cells to DTX (0.01 nM), Tel (0.1 µM) and ADH‐1 (40 µM) caused a significant increase in the number of apoptotic cells compared with the control cells (*p* < 0.001). In addition, treatment of PC3 cells with 40 µM of ADH‐1 had more effect on induction of early apoptosis compared with DTX (Figure 5, *p* < 0.001). Treatment of DU145 cells with abovementioned concentrations of DTX, Tel and ADH‐1 caused a significant increase in the number of apoptotic cells compared with the control cells (*p* < 0.01). However, DTX was more effective on induction of apoptosis compared with Tel and ADH‐1 in this cell line (Figure 5, *p* < 0.01 and *p* < 0.05 respectively).

The exposition of MDA‐MB‐468 cells to DTX, Tel and ADH‐1 caused a significant increase in the number of apoptotic cells compared with the control cells (Figure 5, *p* < 0.05).

## 4 DISCUSSION

In the present investigation, PC3 and MDA‐MB‐468 cell lines which are positive for expression of N‐cadherin were studied and DU145 cell line which does not express N‐cadherin was used as a negative control to survey the specificity of Tel effects on N‐cadherin related cell behaviours. Experiments exploring N‐cadherin specific interaction on Tel were compared to ADH‐1, as an approved N‐cadherin antagonist.

We observed the cytotoxic effect of Tel and DTX on DU145, and PC3 as well as MDA‐MB‐468 cell line. Similarly, ADH‐1, reduced the cell proliferation in all three cell lines. Consistent with our results, treatment of neuroblastoma cell lines with ADH‐1 has shown the strong suppression of tumour cell proliferation by activating apoptosis which in turn indicated N‐cadherin signalling involvement in neuroblastoma development.^29^ Previous studies have revealed that cell‐cell adhesion mediated by cadherins, activates cell survival signals. Although these survival signals are not completely understood, several pathways have been reported to be activated by cadherin‐mediated cell–cell contacts, including Wnt and receptor tyrosine kinase signalling. Administration of ADH‐1 in animal model of pancreatic cancer, demonstrated a significant inhibitory effect on tumour growth and metastasis.^12^ Importantly, the results of a phase I/II clinical trial has shown combination of ADH‐1 and Melphalan (an antineoplastic drug) could suppress tumour growth in patients with advanced melanoma.^30^ Melphalan, acts as DNA alkylating agent that leading to the formation of mono adducts with cross‐links in DNA. It can cause deformity in DNA by cross‐linking and preventing the separation of double‐stranded DNA for the synthesis or transcription that leading to cell apoptosis.^31^ In agreement with our results, Matsuyama et al.^19^ reported that Tel induced inhibitory effect on cell proliferation in PC3 and DU145 cells 48 hours post treatment but other angiotensin II type 1 receptor blockers such as Candesartan and Valsartan have no significant effects on cell viability. Some researchers demonstrated Tel, by inducing S‐phase and G_0_/G_1_ phase arrest and decreasing cyclin D_1_, inhibited cell proliferation in esophageal squamous carcinoma and cholangiocarcinoma.^20^, ^32^ In addition, recent studies have shown that Tel inhibits the proliferation and migration of cancer cells in many cancer types *in vitro* and tumour growth in vivo.^33^, ^34^ Therefore, Tel may disrupt cell–cell contacts by inhibiting N‐cadherin and promotes apoptosis in cancer cells similar to ADH‐1. The homophilic attachment of N‐cadherin extracellular domains between adjacent cells leads to organization of actin cytoskeletal structure and prepares specific outside‐in signals that caused to stability in Bcl‐2 as a member of antiapoptotic proteins and subsequent suppression of cell apoptosis. Therefore, inhibition of homophilic linkage between the N‐cadherin extracellular domains by an antagonist results in a decreasing in Bcl‐2 protein expression and induction of apoptosis in cancerous cells.^23^

Although both Tel and ADH‐1 reduced the cell viability of cancer cells, there was a small difference in the effect of these drugs on PC3, DU145 and MDA‐MB‐468 cell lines. So that, both drugs have more cytotoxic effects on PC3 and MDA‐MB‐468 cell lines compared with DU145 cells, which is probably due to the difference in expression of N‐cadherin. But the effects of DTX as a chemotherapeutic agent in these cell lines were almost the same. Overall, the mechanisms involved in the heterogeneous responses were seen in different cell lines in the current study, which need further investigation. Previous studies have shown that Tel is a partial agonist for PPAR‐γ and the intensity of PPAR‐γ expression in cancerous cells and tissues was higher than their normal counterparts, and the use of its ligands (Tel) induces apoptosis in cancer cells.^17^, ^19^, ^21^ It seems that Tel not only inhibits cell proliferation through PPAR‐γ and cell cycle, but also shows antitumor effects by inhibiting N‐cadherin function. It may explain similar anti‐proliferative effect of Tel on both N‐cadherin expressing and non‐expressing cells.

By promoting the capacity of tumour cell migration and invasion, N‐cadherin has been implicated in various cancers metastasis. PCa typically has no clear symptoms in its early stages but it is often fatal because of the high migratory nature of its cells.^35^ Scratch wound assay was used in the present study to evaluate the effect of Tel as an N‐cadherin antagonist and DTX on PCa and breast cancer cell migration. As expected, Tel inhibited the cell migration rate in PC3 cells and 0.1 µM of Tel has more inhibitory effect than other doses after 24 hours (*p* < 0.001). Besides, both DTX and Tel could decrease the migration of MDA‐MB‐468 cells compared to untreated cells after 24 hours (*p* < 0.001). However, treating DU145 cells with Tel indicated different results, so that Tel had no significant effect on wound closure after 24 hours but DTX inhibited cell migration significantly. Previous studies reported that DTX capable to decreases endothelial cell motility through intervention with microtubule dynamics, inhibiting Rac1/Cdc42 activation and disrupting the actin cytoskeleton. This is correlated with an inhibitory effect on lamellipodia formation and actin polymerization. For many cellular processes especially cell migration, the correct function of cytoskeleton network, including microtubules, actin filaments and intermediate filaments, is essential.^36^

One of the most important difference between three cell lines in the present study is the higher expression of N‐cadherin as a positive regulator of cancer metastasis in PC3 and MDA‐MB‐468 cell lines compared with DU145 cells.^26^ These results could imply the specificity of Tel inhibitory effect on cell migration through inhibition of N‐cadherin compared with the non‐specific effect of DTX. In fact N‐cadherin contribution on generation of cell–cell junctions is less than that of E‐cadherin, and its higher expression in PC3 cells is closely related to ‘more mesenchymal phenotype’ and increased cell invasion capacity of these cells compared with DU145 cells.^37^ Based on our previous in silico studies,^15^, ^16^ we hypothesized that Tel could act as N‐cadherin antagonist. Therefore, in contrast to DU145 cells, Tel inhibited migratory behaviour of PC3 and MDA‐MB‐468 cells. In accordance with our observations, treatment of breast cancer cell lines (MCF7 and MDA‐MB‐231 cells) with 10 µM of Tel reduced the wound closure rate after 24 hours.^38^ In addition, Zhang et al.^39^ reported that treatment of A549 cells (lung cancer) with Tel as an angiotensin receptor blocker could suppress cell proliferation and migration. There are many studies about the effect of Tel on inhibition of cell proliferation and migration, and the mechanisms of Tel function have been stated as an angiotensin receptor blocker or PPAR‐γ agonist. However, none of them have investigated the effect of Tel on N‐cadherin inhibition.^19^, ^21^, ^38^, ^40^ In the present study for the first time, a new mechanism is suggested for Tel by which exerts its functions.

Upon EMT, in which E‐cadherin switches to N‐cadherin, tumour cells tend to detach from their primary location. After invasion into blood vessels, the tumour cells avoid from anoikis process due to their aggregation and attachment to endothelial cells, which is mediated by adhesion molecules such as N‐cadherin.^35^ Experiments on specific blocking behaviour of N‐cadherin confirmed that ADH‐1 significantly reduced the percentage of cell attachment compared with the control cells (*p* < 0.01) and surprisingly, Tel had a significant effect on cell attachment inhibition in PCa and MDA‐MB‐468 cells. Our data showed that Tel, comparable with ADH‐1 reduced the percentage of cell attachment near to 60% in PC3 and MDA‐MB‐468 cells, while using both ADH‐1 and Tel could decrease cell attachment up to 23% in DU145 cell line.

Mechanistically, to explore the effect of Tel on downstream gene of N‐cadherin signalling pathway, AKT‐1 expression level was studied. The PI3K/AKT signalling pathway was reported to be a fundamental pathway regulating EMT in various cancers.^22^ Our results showed Tel, DTX and ADH‐1 inhibited the expression of AKT‐1 gene at mRNA level in PC3, DU145 and MDA‐MB‐468 cells (*p* < 0.01). Therefore, it could be inferred that in addition to inhibiting N‐cadherin, Tel and ADH‐1 act as inhibitors of AKT‐1 in these cell lines, as well. In agreement with our results, Singh and colleagues demonstrated that treatment of DU145 and C4‐2B cells with combination of DTX and Thymoquinone (a phytochemical of black cumin) reduced the expression level of PI3K and AKT‐1.^41^ There are several mechanisms by which Thymoquinone could exert its anticancer activity including, inhibition of JAK/STAT signalling pathway, epidermal growth factor receptor, Wnt signalling pathway and activation of PTEN, P53, caspase‐3,9 as well as induction of reactive oxygen species production that resulting in DNA damage. In general, Thymoquinone via these actions can induce cancer cell apoptosis.^42^ Zhang et al.^39^ reported that treatment of A549 cells, a lung cancer cell line, with 20 μM of Tel significantly decreased the phosphorylated form of AKT‐1 but had no significant effect on the total protein level of AKT‐1. The difference among our data and those of Zhang et al. may be because of using the cancer cell lines from different tissues in the studies. Some researchers indicated that 10 µM of Tel or 2 µM of this drug in combination with 2 µM of Gefitinib, a selective inhibitor of epidermal growth factor receptor tyrosine kinase, via upregulation of phosphatase and tensin homologue (PTEN) transcription can inhibit the PI3K/AKT pathway in cancer cells.^43^, ^44^ To inhibit the PI3K/AKT pathway, novel agents have been developed and most of which are small molecules with reported adverse effects such as hyperglycaemia, insulin resistance and mood alterations.^45^, ^46^ While Tel has significant efficacy and low side effects with a good tolerability when administrates as an antihypertensive drug.^47^

Our results demonstrated treatment of PCa and breast cancer cells with DTX, Tel and ADH‐1 increased the population of dead apoptotic cells compared with the control cells after 48 hours incubation. As seen, Tel and ADH‐1 have the same or more effects on induction of early apoptosis in PC3 and MDA‐MB‐468 cell lines compared with the effect of DTX, while in DU145 cells DTX has more effect on cell apoptosis compared with Tel and ADH‐1. In line with our results, Shintani et al.^12^ indicated that ADH‐1 induces apoptosis in endothelial cells by inhibiting cadherin‐mediated activation of fibroblast growth factor receptors (FGFR) signalling. Other researchers also, reported Tel and DTX could promote apoptosis in different cancer cell lines.^19^, ^36^, ^40^, ^48^

The fact that Tel not only inhibited cell attachment and cell migration but also suppression of AKT‐1 expression, reduced cell proliferation and increased apoptosis in PC3 and MDA‐MB‐468 cell lines, indicates the potential of this small molecule to exert its multimodal antitumor effects in vitro.

In conclusion, based on our results it could be concluded that Tel, as a small molecule, is specifically interact with N‐cadherin and it might be the mechanism by which Tel exerts its effects in addition to the previously reported mechanisms such as its partial agonist role for PPAR‐γ. This study highly implicates N‐cadherin as a valid target for treatment of human PCa, and suggests that N‐cadherin antagonists such as ADH‐1 and Tel that target its adhesive function should be developed for use in treatment of human PCa. Results of the present investigation could suggest Tel as a less expensive alternative to ADH‐1 which potentiate DTX anticancer effects, although many functions and interactions of Tel inhibitory effects on N‐cadherin needs to be explored *in vivo* in the future studies.

## CONFLICT OF INTEREST

The authors confirm that there are no conflicts of interest.

## AUTHOR CONTRIBUTIONS

**Marjan Khorsand:** Investigation (equal); Methodology (equal); Project administration (equal); Writing – original draft (equal). **Sahar Khajeh:** Conceptualization (equal); Investigation (equal); Writing – review & editing (equal). **Mahboobeh Eslami:** Conceptualization (equal); Investigation (equal). **Navid Nezafat:** Conceptualization (equal); Investigation (equal). **Younes Ghasemi:** Conceptualization (equal); Investigation (equal); Project administration (equal). **Vahid Razban:** Conceptualization (equal); Investigation (lead); Project administration (lead); Supervision (lead); Writing – review & editing (lead). **Zohreh Mostafavi‐Pour:** Conceptualization (lead); Investigation (lead); Methodology (lead); Supervision (lead); Writing – review & editing (lead).

## Data Availability

The data that support the findings of this study are available within the article.
